# Breakfast Dietary Pattern Is Inversely Associated with Overweight/Obesity in European Adolescents: The HELENA Study

**DOI:** 10.3390/children8111044

**Published:** 2021-11-12

**Authors:** Leandro Teixeira Cacau, Pilar De Miguel-Etayo, Alba M. Santaliestra-Pasías, Natalia Giménez-Legarre, Dirce Maria Marchioni, Cristina Molina-Hidalgo, Laura Censi, Marcela González-Gross, Evangelia Grammatikaki, Christina Breidenassel, Thaïs De Ruyter, Mathilde Kersting, Frederic Gottrand, Odysseas Androutsos, Sonia Gómez-Martinez, Anthony Kafatos, Kurt Widhalm, Peter Stehle, Dénes Molnár, Yannis Manios, Stefaan De Henauw, Luis A. Moreno

**Affiliations:** 1 Department of Nutrition, School of Public Health, University of São Paulo, São Paulo 01246-904, Brazil; marchioni@usp.br; 2 Growth, Exercise, Nutrition and Development (GENUD) Research Group, Facutlad de Ciencias de la Salud, Universidad de Zaragoza, 50009 Zaragoza, Spain; pilardm@unizar.es (P.D.M.-E.); albasant@unizar.es (A.M.S.-P.); nglegarre@unizar.es (N.G.-L.); lmoreno@unizar.es (L.A.M.); 3 Centro de Investigación Biomédica en Red de Fisiopatología de la Obesidad y Nutrición (CIBEROBN), Instituto de Salud Carlos III, 28040 Madrid, Spain; marcela.gonzalez.gross@upm.es; 4 Instituto Agroalimentario de Aragon (IA2), 50013 Zaragoza, Spain; 5 Instituto de Investigacion Sanitaria Aragon (IIS Aragon), 50009 Zaragoza, Spain; 6 Department of Psychology, University of Pittsburgh, Pittsburgh, PA 15260, USA; molinac@ugr.es; 7 Research Centre for Food and Nutrition, Council for Agricultural Research and Economics (CREA), 00178 Rome, Italy; laura.censi@crea.gov.it; 8 ImFINE Research Group, Department of Health and Human Performance, Universidad Politécnica de Madrid, 28040 Madrid, Spain; breidenassel@dge.de; 9 Department of Nutrition and Dietetics, School of Health Science and Education, Harokopio University, 17671 Athens, Greece; evagram@hua.gr (E.G.); oandrou@hua.gr (O.A.); manios@hua.gr (Y.M.); 10 Department of Public Health and Primary Care, Ghent University, P/A UZ 4K3, Corneel Heymanslaan 10, B-9000 Ghent, Belgium; thais.deruyter@ugent.be (T.D.R.); stefaan.dehenauw@ugent.be (S.D.H.); 11 Department of Nutrition and Food Sciences, University of Bonn, D-53115 Bonn, Germany; p.stehle@uni-bonn.de; 12 Research Department of Child Nutrition, University Hospital of Pediatrics and Adolescent Medicine, Ruhr-University Bochum, D-44791 Bochum, Germany; mathilde.kersting@ruhr-uni-bochum.de; 13 Institute for Translational Research in Inflammation (INFINITE), University Lille, CHU Lille, U1286, F-59000 Lille, France; frederic.gottrand@chru-lille.fr; 14 Department of Nutrition and Dietetics, University of Thessaly, 42100 Trikala, Greece; 15 Immunonutrition Group, Department of Metabolism and Nutrition, Institute of Food Science, Technology and Nutrition (ICTAN), Spanish National Research Council (CSIC), 28040 Madrid, Spain; sgomez@ictan.csic.es; 16 Faculty of Medicine, University of Crete, Crete, 71500 Heraklion, Greece; kafatos@med.uoc.gr; 17 Department of Gastroenterology and Hepatology, Medical University of Vienna, 1090 Vienna, Austria; kurt.widhalm@meduniwien.ac.at; 18 Department of Pediatrics, Medical School, University of Pécs, H-7624 Pécs, Hungary; denes.molnar@aok.pte.hu; 19 Institute of Agri-Food and Life Sciences, Hellenic Mediterranean University Research Centre, 71410 Heraklion, Greece

**Keywords:** dietary patterns, obesity, adolescents, breakfast

## Abstract

Obesity in children and adolescents is a public health problem and diet can play a major role in this condition. We aimed to identify sex-specific dietary patterns (DP) and to evaluate the association with overweight/obesity in European adolescents. We conducted a cross-sectional analysis with 2327 adolescents aged between 12.5 to 17.5 years from a multicenter study across Europe. The body mass index was categorized in “normal weight” and “overweight/obesity”. Two non-consecutive 24-h dietary recalls were collected with a computerized self-reported software. Principal component factor analysis was used to identify DP. Mixed-effect logistic regression models were used to evaluate the association between the sex-specific DP and overweight/obesity outcome. As a result, we found three DP in boys (snacking and bread, Mediterranean diet, and breakfast) and four DP in girls (convenience, plant-based and eggs, Western, and breakfast). The association between DP and overweight/obesity highlights that those adolescents with higher adherence to the breakfast DP had lower odds for overweight/obesity, even after the inclusion of covariables in the adjustments. In European adolescents, the breakfast DP positively characterized by breakfast cereals, fruit, milk, and dairy and negatively characterized by sugar-sweetened beverages in boys and negatively characterized by cereals (pasta, rice, and others) in girls, was inversely associated with overweight/obesity.

## 1. Introduction

Worldwide, the prevalence of obesity in children and adolescents aged between 5–19 years is a serious public health issue, with an increase of about 20% of prevalent cases between the years 1975 to 2016 [1]. Besides being correlated with a higher risk of chronic non-communicable diseases such as diabetes mellitus type 2, hypertension, or metabolic syndrome [2], childhood obesity is also related to adverse consequences in terms of psychological problems and lower educational attainment [3,4].

Several factors are known to influence the risk to develop childhood obesity, including socioeconomic, behavioral, mental, environmental, hereditary, sedentarism, and dietary habits [5], demonstrating the complexity and multifactorial process of this serious health problem. Furthermore, diet is a complex exposure and has several determinants [6]; therefore, the use of a technique capable of evaluating diets in a holistic way seems to be the most appropriate to offer new comprehension into the interrelation between diet and obesity. Best known approaches are a posteriori dietary patterns or multivariate statistical methods, which consists of a data reduction technique with the aim of summarizing the variation in food intakes into a small number of patterns or clusters [7,8]. The most frequently used exploratory statistical methods to derive dietary patterns are cluster analysis, factor analysis (FA), and principal component analysis (PCA) [7,8,9].

Previous studies have used these methods to identify dietary patterns and their relationship with nutritional status in adolescents [10,11,12,13]. A recent review shows that a dietary patterns generally characterized by foods rich in sodium, fats, refined carbohydrates, and low in fiber, such as processed meats, fast-foods, confectionery, refined grains and sugar-sweetened beverages (SSBs) were associated with increased body weight, while a dietary pattern generally characterized by fruit, vegetables, whole grains, and white meats had a positive influence on body weight and adiposity in adolescents [14]. However, the evidence of the relationship between dietary patterns and obesity in adolescents is still uncertain, and more studies on this theme are needed to clarify the role of diet in obesity development in children and adolescents. Besides that, some studies have identified dietary patterns without considering sex-specificities [10,14], which can lead to inappropriate estimates, as there is evidence of different eating habits and prevalence of overweight/obesity in adolescents [15,16]. Thus, this study aimed to (1) derive sex-specific dietary patterns of European adolescents, and (2) evaluate the association between these dietary patterns with the overweight/obesity outcome in a well-established multicenter study in Europe.

## 2. Materials and Methods

### 2.1. Study Design and Population

This is a cross-sectional study that used data from the Healthy Lifestyle in Europe by Nutrition in Adolescence Cross-Sectional Study (HELENA–CSS), a multicenter study conducted in ten European cities (Ghent in Belgium; Dortmund in Germany; Lille in France; Heraklion in Crete; Zaragoza in Spain; Athens in Greece; Pécs in Hungary; Rome in Italy; Stockholm in Sweden; and Vienna in Austria) between 2006 and 2007 with adolescents aged between 12.5 to 17.5 years. Details of the sample, objectives and data collection methods of this study were previously published [17,18]. Briefly, the HELENA–CSS was designed to obtain reliable and comparable data from European adolescent from these cities using nutrition and health-related parameters, such as dietary data, choice and preferences of food, anthropometric, physical fitness and activity, status parameters of vitamin, mineral, lipid and glucose metabolism, and genetic indicators [17,19]. Finally, a total of 3528 (1845 females) adolescents participated in the study [17,18]. Since no 24-h dietary recalls (24H-DRs) were available from adolescents in Pécs (Hungary) and Heraklion (Greece), these cities were not considered in the present evaluation. Therefore, only adolescents with two 24H-DRs were included (final study size: *n* = 2327; 53.8% females).

The HELENA–CSS study was conducted according to the ethical guidelines of the Declaration of Helsinki 1964 (revision of 2000) and good clinical practice. The study was approved by the Research Ethics Committees of each study center with the ethical approval code from the coordinator center 03/2006. All participants and their parents or guardians signed an informed consent [20].

### 2.2. Anthropometric Measurements

Anthropometric measurements were performed by trained researchers following standard protocols [21]. Height was measured to the nearest 0.1 cm using a telescopic height-measuring instrument (model 225; SECA, Hamburg, Germany). Body weight was measured to the nearest 0.1 kg using an electronic scale (model 871; SECA, Hamburg, Germany). All measurements were performed in underwear and barefoot [21].

The BMI was calculated by dividing body weight in kilograms by squared body height in meters (kg/m^2^) according to age and sex as proposed by Cole et al. [22]. Adolescents with BMI < 25 kg/m^2^ were classified as “normal weight” and those with BMI ≥ 25 kg/m^2^ as “overweight/obesity”, based on the international BMI criteria for adolescents [22].

### 2.3. Dietary Assessment

Dietary data were obtained using the HELENA Dietary Assessment Tool (HELENA-DIAT), a software developed for the project [23], first validated in Flemish adolescents [24] and then improved and culturally adapted for use among European adolescents, including the addition of national dishes to achieve a European pattern [24]. Briefly, the HELENA-DIAT is a self-administered computerized 24H-DRs. The adolescents completed the HELENA-DIAT on two non-consecutive days in a period of two weeks. This method has been used and recommended to assess dietary intake in European children and adolescent [25]. Besides that, a trained dietitian was present to support the adolescents in case they needed any clarification to complete the HELENA-DIAT [18].

To calculate energy intake, data from HELENA-DIAT were linked to the German Food Code and Nutrient Database (Bundeslebensmittelschlüssel, vII.3.1, Karlsruhe, Germany) [26]. To remove the intrapersonal variance and to calculate the individual usual consumption, the Multiple Source Method (MSM) was used [18]. This method consists of a statistical program available online to estimate the usual consumption of nutrients and foods. First the individuals’ dietary intake was calculated and based on these data, the population consumption distribution was built [27,28].

The reported foods were then classified into the following 26 food and beverages groups: breads, breakfast cereals, cereals (pasta, rice, and other cereals), bakery products, snacks, sugar (sugar, honey, and other sugars products), vegetables oils, nuts and seeds, butter and margarine, sauces, pulses, vegetables, tubers, fruit, soups, coffee and tea, juices, sugar-sweetened beverages (SSBs), alcohol, meat, fish, eggs, milk, dairy, cheese, mixed foods and desserts.

### 2.4. Physical Activity Measurement

Physical activity was evaluated using the International Physical Activity Questionnaire for Adolescents (IPAQ-A) [29]. The IPAQ-A comprises the following domains: (1) school-related physical activity; (2) transportation; (3) housework and (4) extracurricular physical activity. The number of days per week and the time periods for each day spent walking in moderate and in vigorous physical activity was recorded for each of the IPAQ-A domains [29].

### 2.5. Socioeconomic Status

A modified version of the Family Affluence Scale (FAS) developed by Currie et al. [30] was used as a proxy indicator of socioeconomic status. The FAS considers parameters such as car ownership, having an own bedroom, internet availability, and computer ownership. Adolescents were scored from 0 (lowest) to 8 (highest) and further recategorized into three groups: low (0–2), medium (3–5) and high (6–8) [31].

### 2.6. Statistical Analyses

All statistical analyses were performed using STATA^®^ (Statistical Software for Professionals, College Station, Texas, USA), version 14.2 and the *p-*value < 0.05 was considered statistically significant.

Descriptive analysis was conducted according to sex and the results were presented as mean with their respective 95% confidence intervals (95% CI) for continuous variables and with the number and percentage (*n*, %) for categorial variables. The statistical difference according to sex was evaluated with Student t-test and Pearson’s chi-square test, respectively.

Sex-specific dietary patterns were identified by principal component factor analysis [7]. First, the Kaiser-Meyer-Olkin test (KMO) and the Bartlett’s sphericity test were used to assess the applicability of the dietary data to the principal component factor analysis. The adequacy of the data was proven when the KMO value > 0.50 and Bartlett *p*-value < 0.05 [9,32]. In our data, for the KMO test, a value of 0.58 was obtained, and *p* < 0.001 for the Bartlett’s sphericity test.

After this procedure, principal component factor analysis was conducted based on the 26 food and beverages groups as input variables. Subsequently, orthogonal varimax rotation was performed to improve the interpretability of dietary patterns. The criteria used to retain the factors were eigenvalues > 1, scree plot shape and interpretability of the factors [9,32].

The food groups were retained in the factor when the factor loading was |>0.30|. Each factor was interpreted and named according to the characteristics of the food and beverages groups with the highest factor loadings. Finally, a factor score was obtained for each sex-specific dietary pattern at the individual level using the “predict” command.

The adherence of each sex-specific dietary pattern was transformed in tertiles, with the 1st tertile indicating lower adherence and with the 3rd tertile indicating higher adherence. Then, mixed-effects logistic regression with a random intercept for study center and adjusted for age, physical activity, FAS and energy intake was conducted to evaluate the association between adherence to sex-specific dietary patterns and overweight/obesity outcome.

## 3. Results

Table 1 presents sex-specific characteristics on age, FAS, BMI, energy intake, and physical activity. The boys had more overweight/obesity (53.7%) than girls (46.3%), with a significant statistical difference (*p* < 0.001).

Principal component factor analysis identified three dietary patterns for boys and four dietary patterns for girls, which explained 22.4% and 25.4% of variance in total dietary intake of this population, respectively. Table 2 shows the sex-specific factor loadings for each dietary pattern, highlighting the food and beverages groups that presented factor loading ≥ |0.3|.

In boys, the first dietary pattern was called “snacking and bread” and was positively characterized by breads, snacks, sugar, butter and margarine, sauces, coffee and tea, SSBs, cheese and desserts; the second dietary pattern was called “Mediterranean diet” and was positively characterized by breads, cereals, vegetables oils, nuts and seeds, pulses, vegetables and cheese, and the third dietary pattern was called “breakfast” and was positively characterized by breakfast cereals, fruit, milk and dairy, and was negatively characterized by SSBs.

In girls, the first dietary pattern was called “convenience” and was positively characterized by breads, cereals, sugar, butter and margarine, sauces, coffee and tea, and cheese; the second dietary pattern was called “plant-based and eggs” and was positively characterized by vegetables oils, nuts and seeds, pulses, vegetables and eggs; the third dietary pattern was called “Western” and was positively characterized by bakery products, snacks, tubers, SSBs, meat and desserts, and negatively characterized by cereals; and finally, the four dietary pattern was called “breakfast” and was positively characterized by breakfast cereals, fruit, milk and dairy and was negatively characterized by cereals.

In the crude regression analysis, the boys in the third tertile of the “snacking and bread” pattern (OR 0.43 95% CI 0.28:0.65; *p* < 0.001), “Mediterranean diet” pattern (OR 0.60 95% CI 0.45:0.90, *p* = 0.014) and “breakfast” pattern (OR 0.66 95% CI 0.45:0.98, *p* = 0.039) had lower odds of overweight/obesity. However, when the models were adjusted for age, physical activity, FAS, and energy intake, only the “breakfast” pattern remained associated with lower odds of overweight/obesity, decreasing to 25% (OR 0.85 95% CI 0.44:0.95, *p* = 0.025) the odds for this outcome in boys in the third tertile of this dietary pattern (Table 3).

For girls, in the crude regression models, those in the second (OR 0.65 95% CI 0.46:0.94, *p* = 0.022) and third (OR 0.63 95% CI 0.43:0.94, *p* = 0.022) tertile of the “Western” pattern and those in the third tertile of the “breakfast” pattern (OR 0.54 95% CI 0.36:0.81, *p* = 0.003) had lower odds for overweight/obesity. Nonetheless, after adjusted models for age, physical activity, FAS, and energy intake, only the “breakfast” pattern remained inversely associated with overweight/obesity, decreasing about 39% the odds for this outcome, in girls in third tertile of this dietary pattern (OR 0.61 95% CI 0.40:0.94, *p* = 0.024) (Table 4).

## 4. Discussion

This analysis of dietary intake data collected in a well-designed study across Europe focused on sex-specific associations between overweight/obesity and dietary patterns in adolescents. This is the first study, to our knowledge, that evaluated sex-specific dietary patterns identified by principal component factor analysis with obesity outcome in this study population. In line with our hypothesis, we found three and four different dietary patterns in boys and in girls, respectively. A significant inverse association with a higher adherence with overweight/obesity was, however, only measurable for the so-called “breakfast” dietary pattern, both in girls and in boys.

Although the dietary patterns identified in the present study are similar to those described in previous evaluations of the HELENA study [33,34,35,36], they show some differences that deserve to be highlighted. First, the dietary patterns analysis is data-driven and population-specific, which means that dietary patterns can be mediated by the population and by the aim of the study [7,8]. Although one of those studies aimed to identify the association between dietary patterns and perceptions of healthy eating [34], others aimed to assess the relationship between sedentary behaviors [36], healthy eating [35], and sociodemographic determinants [33] with dietary patterns.

Borges et al. [33] also found three dietary patterns for boys and four dietary patterns for girls in an analysis using the HELENA database. They found the “breakfast” pattern for boys and girls, which is the same as in our study. In addition, they also found the “Western” pattern for boys and girls, similar to the “Western” pattern found for girls in our study. They also found a dietary pattern called “Traditional European” which was positively characterized by bread and rolls, cereals, vegetable oils, nuts, and seeds, pulses, vegetables, and cheese for boys and positively characterized by vegetables oils, nuts, and seeds, pulses, vegetables, and eggs for girls [33]. We found dietary patterns with factor loadings for these food groups; however, we named “Mediterranean diet” for boys and “plant-based and eggs” for girls, respectively. In the analysis conducted by Gonzaléz-Gil et al. [35], four dietary patterns for boys and five dietary patterns for girls were identified. They also found a “breakfast” pattern characterized by white milk, breakfast cereals, and butter and animal fats in boys and a “healthy breakfast” pattern characterized by fruit, white milk, dairy products, breakfast cereals, and negatively characterized by savory snacks and SSBs in girls, which is similar to the “breakfast” found in our study.

Another study [34] using the HELENA database also found three dietary patterns for boys (Mediterranean, breakfast, and beverages) and four dietary patterns for girls (Mediterranean, breakfast, unhealthy beverages and meat, and healthy snack foods). Although they found a “breakfast” pattern in their study, it differs from that found in ours. In our study, the “breakfast” pattern was positively characterized by breakfast cereals, fruit, milk and dairy, and with negative factor loadings for SSBs in boys and for cereals (pasta, rice and others) in girls, while in the study by Gimenez-Legarre et al. [34] the “breakfast” pattern was characterized by breads and rolls, sugar products, butter and animal fats, margarine and lipids of mixed origins, coffee and tea, and cheese in boys and by breads and rolls, sugar products, margarine and lipids of mixed origins, coffee and tea, and fruit in girls.

Santaliestra-Pasías et al. [36] also found a “breakfast” pattern characterized by breads and rolls, sugar products, margarine and lipids of mixing origins, butter and animal fats, and coffee and tea for boys and girls in HELENA dataset. These differences reinforce that dietary pattern analysis are data-driven and mediated by the aim of the study and the decisions made during the analysis [7,8].

The “breakfast” pattern identified in our study can be considered healthiest, because it has a positive loading for fruit, milk, breakfast cereals and dairy, which are considered healthier foods and being part of a healthy diet. Besides that, this pattern had a negative loading for SSBs in boys and for cereals (pasta, rice and others) in girls. We found an inverse association between higher adherence to this pattern with lower odds for overweight/obesity. Some initiatives have described the importance of regular breakfast consumption for a healthy diet [37,38,39,40,41,42,43] and for health outcomes, including obesity [44,45] and cardiovascular risk factors [46].

However, the associations between the consumption of breakfast and between adherence to an “breakfast” dietary pattern with overweight/obesity in children and adolescents are still uncertain. In a study with Chileans adolescents participants in the Growth and Obesity Chilean Cohort Study (GOCS), Martínez-Arroyo et al. [47] found no associations between the “breakfast” pattern characterized by cold cuts, tea and coffee, bread, sugar, and margarine and butter with obesity outcome, in a prospective analysis. Borges et al. [48] found a “breakfast” pattern similar to the found in our study, characterized by fruit, breakfast cereals, and milk and dairy in a large Brazilian adolescent’s sample, but found no association between the adherence to this pattern with the overweight outcome. However, Howe et al. [49] identified a dietary pattern similar to the “breakfast” of our study, but called “basic foods” characterized by milk, other milks, breakfast cereals and white bread, and they found an inverse association between this dietary pattern with a marker of obesity, the fat mass index (FMI).

Regarding the other dietary patterns found in our results, some studies found associations between adherence to a pattern characterized by high foods rich in sodium, fats, refined carbohydrates and energy density foods with increasing odds for overweight/obesity outcome [14,48,50]. On the other hand, similar to our founds, other studies found no association between these dietary patterns with overweight/obesity in Brazilian [10,51], American [52], and even in European adolescents [11,53]. However, although we did not find a significant association between the patterns characterized by these foods (“snacking and breads” in boys and “convenience” and “Western” in girls), it was possible to find a trend of an association with overweight/obesity for girls, but not for boys. We also did not find a significant association for patterns characterized by plant-based foods, cereals (pasta, rice and other cereals) and eggs (“Mediterranean diet” in boys and “plant-based and eggs” in girls), but we can find a trend of an inverse association with overweight/obesity, after statistical adjustments. One possible explanation for these conflicting results is that obesity is a multifactorial condition, and therefore diet is not the only associated factor. Furthermore, the diet is also a complex exposure, and the analysis of dietary patterns is data-dependent, which makes comparisons between studies difficult.

Nonetheless, our study has some strengths. We used a well-developed large and culturally diverse sample of European adolescents. The food consumption was evaluated using the HELENA-DIAT, an automated tool to collect 24H-DRs which has been described as a good method to obtain dietary data information. We used statistical methods to ensure the quality of the dietary information, such as using the MSM method to remove the intrapersonal variability and using the principal components factor analysis to identify dietary patterns, which can provide a more holistic view of the diet. Besides that, we identified sex-specific dietary patterns due the different diet habits of girls and boys. Furthermore, the data collection of the HELENA study followed standardized procedures and strict protocols.

However, some limitations can be pointed out as well. This is a cross-sectional study, which cannot provide a causal inference between exposure and outcome. As previously mentioned, diet is a complex variable and its assessment is prone to errors, since the methods of assessment of food consumption are self-reported, which depends on the respondent’s memory. Furthermore, despite being a statistical method and providing a more holistic view of the diet, the dietary pattern technique requires a series of arbitrary decisions. The HELENA study was carried out between 2006 and 2007 and despite including a large sample size it is still not representative of all adolescents, such those living in rural regions. The results of this study contributing to the field of nutritional epidemiology, through the analysis of overweight/obese and dietary patterns in adolescents.

## 5. Conclusions

In conclusion, our results showed that a sex independent “breakfast” pattern exhibits a protective effect against overweight/obesity in adolescents, even after controlling for potential confounding variables. Therefore, our results highlight that a healthier breakfast pattern can play a role in preventing obesity in adolescents. Furthermore, studies that focus on the breakfast quality with odds for obesity may be of interest, especially prospective studies.

## Figures and Tables

**Table 1:** Characteristics of the study population according to sex. HELENA study.

	Boys (*n* = 1075)	Girls (*n* = 1252)	*p*-Value
Age (years), mean (95% CI)	14.8 (14.7:14.9)	14.7 (14.6:14.7)	0.096
FAS, *n (%)*			0.081
Low	101 (39.6)	154 (60.4)	
Medium	606 (46.9)	687 (53.1)	
High	362 (47.3)	404 (52.7)	
Overweight/obesity, *n (%)*			<0.001
No	810 (44.2)	1010 (55.8)	
Yes	270 (53.7)	233 (46.3)	
BMI (kg/m^2^), mean (95% CI)	21.3 (21.1:21.6)	21.2 (21.0:21.4)	0.227
Energy intake (kcal), mean (95% CI)	2526.4 (2475.1:2577.8)	1929.6 (1895.8:1963.4)	<0.001
Physical Activity (min/week), mean (95% CI)	1368.1 (1295.6:1440.6)	1196.6 (1136.5:1256.7)	<0.001

CI: confidence interval. FAS: family affluence scale. BMI: body mass index. Significant values (*p* < 0.05) expressed in bold font.

**Table 2:** Factor loadings of dietary patterns of European adolescents, according to sex. HELENA study.

	Boys			Girls			
Food Groups	*Snacking and Bread*	*Mediterranean Diet*	*Breakfast*	*Convenience*	*Plant-Based and Eggs*	*Western*	*Breakfast*
Breads	**0.58**	**0.30**	0.09	**0.63**	0.12	0.17	0.04
Breakfast cereals	0.17	−0.13	**0.55**	0.10	0.04	−0.02	**0.57**
Cereals (pasta, rice, and other)	0.09	**0.46**	−0.07	**0.36**	0.26	**−0.30**	**−0.35**
Bakery products	−0.04	0.27	0.06	−0.04	0.18	**0.31**	−0.09
Snacks	**0.38**	−0.02	−0.21	−0.05	0.20	**0.47**	−0.22
Sugar (sugar, honey, and other)	**0.32**	0.01	0.24	**0.44**	0.05	0.01	0.15
Vegetables oils, nuts and seeds	−0.03	**0.76**	−0.09	−0.04	**0.72**	−0.03	0.01
Butter and margarine	**0.56**	−0.06	0.22	**0.62**	−0.19	0.06	0.17
Sauces	**0.38**	−0.04	−0.11	**0.30**	−0.13	0.19	−0.05
Pulses	−0.18	**0.40**	0.05	−0.20	**0.30**	−0.06	0.13
Vegetables	0.08	**0.63**	0.15	0.09	**0.62**	0.01	0.07
Tubers	0.17	−0.24	0.18	−0.04	−0.09	**0.40**	0.28
Fruit	0.04	0.19	**0.42**	0.19	0.12	0.03	**0.47**
Soups	−0.01	0.01	0.26	−0.16	0.09	0.25	0.05
Coffee and tea	**0.34**	0.03	−0.20	**0.43**	−0.06	0.02	0.05
Juices	0.21	−0.03	0.05	0.07	−0.02	0.17	0.05
SSBs	**0.46**	−0.13	**−0.42**	0.18	−0.08	**0.60**	−0.10
Alcohol	0.20	0.04	−0.27	−0.01	−0.01	0.15	−0.21
Meat	0.22	0.19	0.11	0.06	0.08	**0.33**	0.08
Fish	−0.20	0.15	0.25	−0.18	0.25	−0.09	0.15
Eggs	0.04	0.15	0.17	0.05	**0.41**	0.03	0.09
Milk	−0.03	−0.03	**0.70**	0.04	0.13	−0.21	**0.58**
Dairy	0.10	−0.18	**0.30**	−0.02	−0.18	0.20	**0.33**
Cheese	**0.36**	**0.47**	−0.07	**0.45**	0.26	−0.07	−0.28
Mixed foods	−0.02	−0.05	−0.19	−0.16	0.13	0.03	−0.15
Desserts	**0.49**	−0.03	−0.08	0.15	−0.05	**0.53**	0.01
% variance	7.8	7.7	6.9	7.1	6.2	6.2	5.9
% cumulative	7.8	15.5	22.4	7.1	13.3	19.5	25.4

SSBs: sugar-sweetened beverages. In bold factor loadings |>0.30|.

**Table 3:** Association between the tertiles of dietary patterns and overweight/obesity in the boys. HELENA study.

	Crude			Adjusted£		
	OR	95% CI	*p*-Value	OR	95% CI	*p*-Value
*Snacking and bread dietary pattern*						
1st tertile	ref					
2nd tertile	0.71	0.49:1.03	0.071	0.90	0.60:1.35	0.600
3rd tertile	**0.43**	**0.28:0.65**	**<0.001**	0.80	0.46:1.39	0.436
*Mediterranean diet dietary pattern*						
1st tertile	ref					
2nd tertile	0.77	0.54:1.11	0.165	0.83	0.57:1.22	0.353
3rd tertile	**0.60**	**0.40:0.90**	**0.014**	0.93	0.58:1.49	0.772
*Breakfast dietary pattern*						
1st tertile	ref					
2nd tertile	0.86	0.61:1.23	0.416	0.90	0.61:1.31	0.567
3rd tertile	**0.66**	**0.45:0.98**	**0.039**	**0.85**	**0.44:0.95**	**0.025**

OR: odds ratio. 95% CI: 95% confidence interval. Adjusted for age, physical activity, FAS, and energy intake.

**Table 4:** Association between the tertiles of dietary patterns and overweight/obesity in the girls. HELENA study.

	Crude			Adjusted£		
	OR	95% CI	*p*-Value	OR	95% CI	*p*-Value
Convenience dietary pattern						
1st tertile	Ref					
2nd tertile	0.86	0.59:1.24	0.414	1.16	0.78:1.75	0.463
3rd tertile	0.74	0.49:1.10	0.133	1.38	0.86:2.20	0.181
Plant-based and eggs dietary pattern						
1st tertile	Ref					
2nd tertile	0.86	0.59:1.24	0.411	0.98	0.65:1.46	0.912
3rd tertile	0.61	0.39:0.95	0.029	0.84	0.51:1.38	0.496
Western dietary pattern						
1st tertile	Ref					
2nd tertile	0.65	0.46:0.94	0.022	0.82	0.56:1.22	0.333
3rd tertile	0.63	0.43:0.94	0.022	1.17	0.73:1.87	0.521
Breakfast dietary pattern						
1st tertile	Ref					
2nd tertile	0.87	0.61:1.24	0.451	0.74	0.51:1.07	0.118
3rd tertile	0.54	0.36:0.81	0.003	0.61	0.40:0.94	0.024

OR: odds ratio. 95% CI: 95% confidence interval. Adjusted for age, physical activity, FAS, and energy intake.

## References

[1] NCD Risk Factor Collaboration (NCD-RisC) (2017). Worldwide trends in body-mass index, underweight, overweight, and obesity from 1975 to 2016: A pooled analysis of 2416 population-based measurement studies in 128·9 million children, adolescents, and adults. Lancet.

[2] Park M.H., Falconer C., Viner R.M., Kinra S. (2012). The impact of childhood obesity on morbidity and mortality in adulthood: A systematic review. Obes. Rev. Off. J. Int. Assoc. Study Obes..

[3] Pulgarón E.R. (2013). Childhood obesity: A review of increased risk for physical and psychological comorbidities. Clin. Ther..

[4] Rankin J., Matthews L., Cobley S., Han A., Sanders R., Wiltshire H.D., Baker J.S. (2016). Psychological consequences of childhood obesity: Psychiatric comorbidity and prevention. Adolesc. Health Med. Ther..

[5] Rolland-Cachera M.F. (2011). Childhood obesity: Current definitions and recommendations for their use. Int. J. Pediatric Obes. IJPO Off. J. Int. Assoc. Study Obes..

[6] Satija A., Stampfer M.J., Rimm E.B., Willett W., Hu F.B. (2018). Perspective: Are Large, Simple Trials the Solution for Nutrition Research?. Adv. Nutr..

[7] Hu F.B. (2002). Dietary pattern analysis: A new direction in nutritional epidemiology. Curr. Opin. Lipidol..

[8] Ocké M.C. (2013). Evaluation of methodologies for assessing the overall diet: Dietary quality scores and dietary pattern analysis. Proc. Nutr. Soc..

[9] Santos R., Gorgulho B., Castro M., Fisberg R., Marchioni D., Baltar V. (2019). Principal Component Analysis and Factor Analysis: Differences and similarities in Nutritional Epidemiology application. Rev. Bras. Epidemiol..

[10] De Almeida Alves M., Retondario A., Bricarello L.P., Fernandes R., de Moura Souza A., Zeni L.A.Z.R., de Moraes Trindade E.B.S., de Assis Guedes de Vasconcelos F. (2020). Association between dietary patterns and overweight/obesity: A Brazilian national school-based research (ERICA 2013–2014). J. Public Health.

[11] Huybrechts I., Lioret S., Mouratidou T., Gunter M.J., Manios Y., Kersting M., Gottrand F., Kafatos A., De Henauw S., Cuenca-García M. (2017). Using reduced rank regression methods to identify dietary patterns associated with obesity: A cross-country study among European and Australian adolescents. Br. J. Nutr..

[12] Song Y., Park M.J., Paik H.Y., Joung H. (2010). Secular trends in dietary patterns and obesity-related risk factors in Korean adolescents aged 10–19 years. Int. J. Obes..

[13] Ambrosini G.L., Huang R.C., Mori T.A., Hands B.P., O’Sullivan T.A., de Klerk N.H., Beilin L.J., Oddy W.H. (2010). Dietary patterns and markers for the metabolic syndrome in Australian adolescents. Nutr. Metab. Cardiovasc. Dis. NMCD.

[14] Neves M.E.A., Souza M.R.d., Gorgulho B.M., Cunha D.B., Muraro A.P., Rodrigues P.R.M. (2021). Association of dietary patterns with blood pressure and body adiposity in adolescents: A systematic review. Eur. J. Clin. Nutr..

[15] Badr H.E., Lakha S.F., Pennefather P. (2019). Differences in physical activity, eating habits and risk of obesity among Kuwaiti adolescent boys and girls: A population-based study. Int. J. Adolesc. Med. Health.

[16] Askovic B., Kirchengast S. (2012). Gender differences in nutritional behavior and weight status during early and late adolescence. Anthropol. Anz. Ber. Über Die Biol.-Anthropol. Lit..

[17] Moreno L.A., De Henauw S., González-Gross M., Kersting M., Molnár D., Gottrand F., Barrios L., Sjöström M., Manios Y., Gilbert C.C. (2008). Design and implementation of the Healthy Lifestyle in Europe by Nutrition in Adolescence Cross-Sectional Study. Int. J. Obes..

[18] Moreno L.A., Gottrand F., Huybrechts I., Ruiz J.R., González-Gross M., DeHenauw S. (2014). Nutrition and lifestyle in european adolescents: The HELENA (Healthy Lifestyle in Europe by Nutrition in Adolescence) study. Adv. Nutr..

[19] De Henauw S., Gottrand F., De Bourdeaudhuij I., Gonzalez-Gross M., Leclercq C., Kafatos A., Molnar D., Marcos A., Castillo M., Dallongeville J. (2007). Nutritional status and lifestyles of adolescents from a public health perspective. The HELENA Project—Healthy Lifestyle in Europe by Nutrition in Adolescence. J. Public Health.

[20] Béghin L., Castera M., Manios Y., Gilbert C.C., Kersting M., De Henauw S., Kafatos A., Gottrand F., Molnar D., Sjöström M. (2008). Quality assurance of ethical issues and regulatory aspects relating to good clinical practices in the HELENA Cross-Sectional Study. Int. J. Obes..

[21] Nagy E., Vicente-Rodriguez G., Manios Y., Béghin L., Iliescu C., Censi L., Dietrich S., Ortega F.B., De Vriendt T., Plada M. (2008). Harmonization process and reliability assessment of anthropometric measurements in a multicenter study in adolescents. Int. J. Obes..

[22] Cole T.J., Bellizzi M.C., Flegal K.M., Dietz W.H. (2000). Establishing a standard definition for child overweight and obesity worldwide: International survey. BMJ (Clin. Res. Ed.).

[23] Vereecken C.A., Covents M., Sichert-Hellert W., Alvira J.M.F., Le Donne C., De Henauw S., De Vriendt T., Phillipp M.K., Béghin L., Manios Y. (2008). Development and evaluation of a self-administered computerized 24-h dietary recall method for adolescents in Europe. Int. J. Obes..

[24] Vereecken C.A., Covents M., Matthys C., Maes L. (2005). Young adolescents’ nutrition assessment on computer (YANA-C). Eur. J. Clin. Nutr..

[25] Andersen L.F., Lioret S., Brants H., Kaic-Rak A., de Boer E.J., Amiano P., Trolle E., EFCOVAL Consortium (2011). Recommendations for a trans-European dietary assessment method in children between 4 and 14 years. Eur. J. Clin. Nutr..

[26] Julián-Almárcegui C., Bel-Serrat S., Kersting M., Vicente-Rodriguez G., Nicolas G., Vyncke K., Vereecken C., De Keyzer W., Beghin L., Sette S. (2016). Comparison of different approaches to calculate nutrient intakes based upon 24-h recall data derived from a multicenter study in European adolescents. Eur. J. Nutr..

[27] Harttig U., Haubrock J., Knüppel S., Boeing H., EFCOVAL Consortium (2011). The MSM program: Web-based statistics package for estimating usual dietary intake using the Multiple Source Method. Eur. J. Clin. Nutr..

[28] Haubrock J., Nöthlings U., Volatier J.L., Dekkers A., Ocké M., Harttig U., Illner A.K., Knüppel S., Andersen L.F., Boeing H. (2011). Estimating usual food intake distributions by using the multiple source method in the EPIC-Potsdam Calibration Study. J. Nutr..

[29] De Cocker K., Ottevaere C., Sjöström M., Moreno L.A., Wärnberg J., Valtueña J., Manios Y., Dietrich S., Mauro B., Artero E.G. (2011). Self-reported physical activity in European adolescents: Results from the HELENA (Healthy Lifestyle in Europe by Nutrition in Adolescence) study. Public Health Nutr..

[30] Currie C., Molcho M., Boyce W., Holstein B., Torsheim T., Richter M. (2008). Researching health inequalities in adolescents: The development of the Health Behaviour in School-Aged Children (HBSC) family affluence scale. Soc. Sci. Med..

[31] Jiménez Pavón D., Ortega F.P., Ruiz J.R., España Romero V., García Artero E., Moliner Urdiales D., Gómez Martínez S., Vicente Rodríguez G., Manios Y., Béghin L. (2010). Socioeconomic status influences physical fitness in European adolescents independently of body fat and physical activity: The HELENA study. Nutr. Hosp..

[32] Cattell R.B. (1966). The Scree Test For The Number Of Factors. Multivar. Behav. Res..

[33] Borges C.A., Slater B., Santaliestra-Pasías A.M., Mouratidou T., Huybrechts I., Widhalm K., Gottrand F., Manios Y., Jimenez-Pavón D., Valtueña J. (2018). Dietary Patterns in European and Brazilian Adolescents: Comparisons and Associations with Socioeconomic Factors. Nutrients.

[34] Giménez-Legarre N., Santaliestra-Pasías A.M., Beghin L., Dallongeville J., de la O.A., Gilbert C., González-Gross M., De Henauw S., Kafatos A., Kersting M. (2019). Dietary Patterns and Their Relationship With the Perceptions of Healthy Eating in European Adolescents: The HELENA Study. J. Am. Coll. Nutr..

[35] González-Gil E., Martínez-Olivan B., Widhalm K., Lambrinou C.-P., De Henauw S., Gottrand F., Kafatos A., Béghin L., Molnár D., Kersting M. (2019). Healthy eating determinants and dietary patterns in European adolescents: The HELENA study. Child Adolesc. Obes..

[36] Santaliestra-Pasías A.M., Mouratidou T., Huybrechts I., Beghin L., Cuenca-García M., Castillo M.J., Galfo M., Hallstrom L., Kafatos A., Manios Y. (2014). Increased sedentary behaviour is associated with unhealthy dietary patterns in European adolescents participating in the HELENA study. Eur. J. Clin. Nutr..

[37] Giménez-Legarre N., Miguel-Berges M.L., Flores-Barrantes P., Santaliestra-Pasías A.M., Moreno L.A. (2020). Breakfast Characteristics and Its Association with Daily Micronutrients Intake in Children and Adolescents–A Systematic Review and Meta-Analysis. Nutrients.

[38] Giménez-Legarre N., Flores-Barrantes P., Miguel-Berges M.L., Moreno L.A., Santaliestra-Pasías A.M. (2020). Breakfast Characteristics and Their Association with Energy, Macronutrients, and Food Intake in Children and Adolescents: A Systematic Review and Meta-Analysis. Nutrients.

[39] O’Neil C.E., Byrd-Bredbenner C., Hayes D., Jana L., Klinger S.E., Stephenson-Martin S. (2014). The role of breakfast in health: Definition and criteria for a quality breakfast. J. Acad. Nutr. Diet..

[40] Forkert E.C.O., Moraes A.C.F.D., Carvalho H.B., Manios Y., Widhalm K., González-Gross M., Gutierrez A., Kafatos A., Censi L., De Henauw S. (2019). Skipping breakfast is associated with adiposity markers especially when sleep time is adequate in adolescents. Sci. Rep..

[41] Gibney M.J., Barr S.I., Bellisle F., Drewnowski A., Fagt S., Livingstone B., Masset G., Varela Moreiras G., Moreno L.A., Smith J. (2018). Breakfast in Human Nutrition: The International Breakfast Research Initiative. Nutrients.

[42] Mielgo-Ayuso J., Valtueña J., Cuenca-García M., Gottrand F., Breidenassel C., Ferrari M., Manios Y., De Henauw S., Widhalm K., Kafatos A. (2017). Regular breakfast consumption is associated with higher blood vitamin status in adolescents: The HELENA (Healthy Lifestyle in Europe by Nutrition in Adolescence) Study. Public Health Nutr..

[43] Moreno Aznar L.A., Vidal Carou M.d.C., López Sobaler A.M., Varela Moreiras G., Moreno Villares J.M. (2021). Papel del desayuno y su calidad en la salud de los niños y adolescentes en España. Nutr. Hosp..

[44] Monzani A., Ricotti R., Caputo M., Solito A., Archero F., Bellone S., Prodam F. (2019). A Systematic Review of the Association of Skipping Breakfast with Weight and Cardiometabolic Risk Factors in Children and Adolescents. What Should We Better Investigate in the Future?. Nutrients.

[45] Szajewska H., Ruszczynski M. (2010). Systematic review demonstrating that breakfast consumption influences body weight outcomes in children and adolescents in Europe. Crit. Rev. Food Sci. Nutr..

[46] Hallström L., Labayen I., Ruiz J.R., Patterson E., Vereecken C.A., Breidenassel C., Gottrand F., Huybrechts I., Manios Y., Mistura L. (2013). Breakfast consumption and CVD risk factors in European adolescents: The HELENA (Healthy Lifestyle in Europe by Nutrition in Adolescence) Study. Public Health Nutr..

[47] Martínez Arroyo A., Corvalán Aguilar C., Palma Molina X., Ceballos Sanchez X., Fisberg R.M. (2020). Dietary Patterns of Adolescents from the Chilean Growth and Obesity Cohort Study Indicate Poor Dietary Quality. Nutrients.

[48] Borges C.A., Marchioni D.M.L., Levy R.B., Slater B. (2018). Dietary patterns associated with overweight among Brazilian adolescents. Appetite.

[49] Howe A.S., Black K.E., Wong J.E., Parnell W.R., Skidmore P.M.L. (2013). Dieting status influences associations between dietary patterns and body composition in adolescents: A cross-sectional study. Nutr. J..

[50] Zhang J., Wang H., Wang Y., Xue H., Wang Z., Du W., Su C., Zhang J., Jiang H., Zhai F. (2015). Dietary patterns and their associations with childhood obesity in China. Br. J. Nutr..

[51] Biazzi Leal D., Altenburg de Assis M.A., Hinnig P.d.F., Schmitt J., Soares Lobo A., Bellisle F., Di Pietro P.F., Vieira F.K., De Moura Araujo P.H., De Andrade D.F. (2017). Changes in Dietary Patterns from Childhood to Adolescence and Associated Body Adiposity Status. Nutrients.

[52] Cutler G.J., Flood A., Hannan P.J., Slavin J.L., Neumark-Sztainer D. (2011). Association between major patterns of dietary intake and weight status in adolescents. Br. J. Nutr..

[53] Richter A., Heidemann C., Schulze M.B., Roosen J., Thiele S., Mensink G.B.M. (2012). Dietary patterns of adolescents in Germany—Associations with nutrient intake and other health related lifestyle characteristics. BMC Pediatr..

